# Molecular characterisation of human rabies in Tanzania and Kenya: a case series report and phylogenetic investigation

**DOI:** 10.1186/s40249-024-01245-w

**Published:** 2024-10-28

**Authors:** Gurdeep Jaswant, Kathryn Campbell, Anna Czupryna, Athman Mwatondo, Brian Ogoti, Carmen W. E. Embregts, Corine H. GeurtsvanKessel, Charles Kayuki, Davis Kuchaka, Gati Wambura, James Oigo, Joel Changalucha, Julius O. Oyugi, Kennedy Lushasi, Lwitiko Sikana, Marco van Zwetselaar, Marieke C. J. Dekker, Mathew Muturi, Marybeth Maritim, Mumbua Mutunga, Rowan Durrant, Tom Abala, Veronicah Chuchu, Kirstyn Brunker, S. M. Thumbi, Katie Hampson

**Affiliations:** 1 Faculty of Health Sciences, Institute of Tropical & Infectious Diseases, University of Nairobi, Nairobi, 00202 Kenya; 2 School of Biodiversity, One Health & Veterinary Medicine, College of Medical, Veterinary & Life Sciences, University of Glasgow, Glasgow, G12 8QQ UK; 3 Food Biotechnology & Microbiology, Tanzania Industrial Research Development Organization, Dar Es Salaam, 14111 Tanzania; 4 Environmental Health & Ecological Sciences, Ifakara Health Institute, Plot 463, Dar Es Salaam, Tanzania; 5 Zoonotic Disease Unit, Ministry of Health and Ministry of Livestock, Nairobi, 00202 Kenya; 6 Center for Epidemiological Modelling & Analysis, University of Nairobi, Nairobi, 00202 Kenya; 7 Viroscience Department, Erasmus MC Rotterdam, Rotterdam, 3015 CN the Netherlands; 8 Oxford Nanopore Technologies, Gosling Building, Edmund Halley Road, Oxford Science Park, Oxford, OX4 4DQ UK; 9 Biotechnology Laboratory, Kilimanjaro Clinical Research Institute, P. O. Box 2236, Moshi, Tanzania; 10 Center for Global Health Research, Kenya Medical Research Institute, Kisumu, 40100 Kenya; 11 Tanzania College of Veterinary Medicine & Biomedical Science, Sokoine University of Agriculture, Morogoro, 67804 Tanzania; 12 Department of Medical Microbiology, Faculty of Health Sciences, University of Nairobi, Nairobi, 00202 Kenya; 13 Department of Clinical Medicine and Therapeutics, University of Nairobi, Nairobi, 00202 Kenya; 14 Paul G Allen School for Global Health, Washington State University, 1155 NE College Ave, Pullman, WA 99164 USA; 15 Institute of Immunology & Infection Research, School of Biological Sciences, University of Edinburgh, Edinburgh, EH9 3FL Scotland, UK

**Keywords:** *Lyssavirus*, One Health, Nanopore, Next-generation sequencing, Whole genome sequencing, Genomic surveillance, East Africa

## Abstract

**Background:**

Rabies remains a major public health problem in low- and middle-income countries. However, human rabies deaths are rarely laboratory-confirmed or sequenced, especially in Africa. Five human rabies deaths from Tanzania and Kenya were investigated and the causative rabies viruses sequenced, with the aim of identifying implications for rabies control at individual, healthcare and societal levels.

**Case presentation:**

The epidemiological context and care of these cases was contrasting. Four had a clear history of being bitten by dogs, while one had an unclear biting history. Two individuals sought medical attention within a day of being bitten, whereas three sought care only after developing rabies symptoms. Despite seeking medical care, none of the cases received complete post-exposure prophylaxis: one patient received only tetanus vaccination, one did not complete the post-exposure vaccination regimen, one followed an off-label vaccination schedule, and two did not receive any post-exposure vaccinations before the onset of symptoms. These cases highlight serious gaps in health-seeking behaviour, and in health systems providing appropriate care following risky exposures, including in the accessibility and effectiveness of post-exposure prophylaxis as it is administered in the region.

**Conclusions:**

The viral genomic and epidemiological data confirms dog-mediated rabies as the cause of each of these deaths. The phylogenetic investigation highlights the transboundary circulation of rabies within domestic dog populations, revealing distinct rabies virus clades with evidence of regional spread. These findings underscore the importance of coordinated cross-border control efforts between the two countries. Urgent action is needed to improve awareness around the need for emergency post-exposure vaccines that should be accessible in local communities and administered appropriately, as well as investment in coordinated dog vaccination to control dog-mediated rabies, the underlying cause of these deaths.

**Graphical Abstract:**

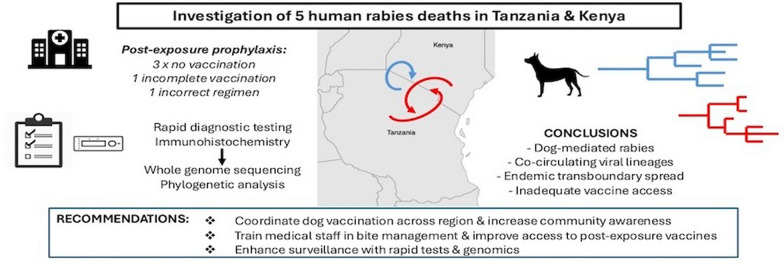

**Supplementary Information:**

The online version contains supplementary material available at 10.1186/s40249-024-01245-w.

## Background

Domestic dogs are the source of the vast majority of human rabies deaths that occur every year globally [1]. In the East Africa region over 2000 human deaths from dog-mediated rabies are estimated to occur annually [2]. Yet human rabies deaths are rarely confirmed from any country in East Africa [3]. The lack of verified statistics documenting the extent of the burden from this notifiable disease contributes to its continued neglect [4]. Unlike in countries, where mass dog vaccination has been used to eliminate rabies and minimise the resulting risk to humans [5, 6], dog vaccination campaigns are not conducted routinely or at scale across East Africa. Moreover, economic constraints, lack of awareness, and logistical challenges hinder access to post-exposure vaccines which are essential to prevent human rabies deaths when the disease still circulates in domestic dog populations [7]. Gavi, the Vaccine Alliance is investing in human rabies vaccines and countries in East Africa are potentially eligible for this support [8]. Improved surveillance of human rabies and understanding of why human rabies deaths still occur could inform Gavi’s support in the region and the countries’ investments towards the global goal of eliminating dog-mediated human rabies deaths.

The rabies virus targets the nervous system, travelling along nerves from the infection site to the brain, where it multiplies and causes rabies [5]. Post-exposure prophylaxis (PEP) is needed as an emergency measure for rabid bite victims to prevent the onset of this rapidly progressive fatal neurologic disease [9]. In the absence of timely PEP, around one in five rabid bite victims will progress to rabies, depending on the site and severity of the bite(s) [10]. The World Health Organization (WHO) recommendations for PEP comprise thorough wound washing followed by a course of post-exposure vaccinations and, in the case of severe exposure, administration of rabies immunoglobulins (RIG) [4]. However, access to PEP and its timely use is not universal. High costs and limited availability of rabies post-exposure vaccines, as well as a lack of awareness about the need for appropriate PEP leads to thousands of otherwise preventable deaths [4, 11, 12].

New approaches for rabies diagnosis such as sensitive molecular methods and sequencing can improve the confirmation of human rabies and be used to characterise pathogen spread [13]. Sequencing approaches have the potential to enhance routine rabies surveillance and provide actionable information to inform elimination programmes, for example, to distinguish whether cases are due to continuous undetected local circulation or from new incursions and to identify the sources of such incursions [14, 15]. More generally, sequencing could provide key insights into how rabies circulates within different populations and the processes responsible for its maintenance in specific geographic localities [16]. In-country genome sequencing of rabies viruses from human rabies cases on the African continent has so far only been carried out in South Africa [3, 17–19], however partial genome sequences are available from one human rabies case from Senegal [20] and one human rabies case from Nigeria [21]. This case series reports rabies virus whole genomes sequenced from five human rabies cases in East Africa, investigating the factors leading to each death and how such deaths might be prevented in future.

## Case presentations

There were no specific selection criteria for choosing the samples included in this study. Rather, the samples were available due to an ongoing surveillance project in these two areas, which provided the necessary dialogue with affected families to enable sample collection and to undertake follow up. All five deaths described resulted from exposures by domestic dogs and a lack of, or inappropriate, PEP administration (Table 1). Four of the five patients were children who were less than 15 years old. Three of the children were bitten on the head or neck, sites that are at highest risk for progression to rabies in the absence of PEP [22].

**Table 1:** Summary of human rabies case histories, diagnostic results and viral genome characterization

	Case 1	Case 2	Case 3	Case 4	Case 5
Sex/Age (year)	Male/10	Male/37	Female/6	Male/6	Male/13
Case location	Nyawara village, Central Gem ward, Gem sub-county, Siaya county, Kenya	Rarieda village, Central Gem ward, Gem sub-county, Siaya county, Kenya	Tarakea-Rombo village, Moshi district, Tanzania	Alara village, South West Sakwa ward, Bondo sub-county, Siaya county, Kenya	Bulati village, Ngorongoro district, Tanzania
Bite history	Bites to the left arm and forehead by an unknown dog	No bite history, but killed his 2 dogs with suspect rabies	Multiple bites to upper lip by an unknown dog	Multiple bites to the head and arm by an unknown dog	Single bite to the left leg by own dog
Exposure date	Approximately 1 month prior to symptoms onset	Unknown	26 Aug 2019	22 June 2022	8 Sep 2022
Delay to attend health facility after exposure	0 day	After symptoms onset (3 July 2018)	0 day	1 day	After symptoms onset (19 days after exposure)
PEP received	Wound cleaning only	None	Wound cleaning; intramuscular vaccine: day 0, 7, 18	Wound cleaning, intramuscular vaccine: day 0 only	Wound cleaning only
Other care at health facility	Paracetamol, anti-tetanus vaccine	None	Paracetamol, anti-tetanus vaccine	None	None
Why no/inadequate PEP?	Not advised	Reported after symptom onset	Received regimen that is not recommended	Not advised	Reported after symptom onset
Incubation period till symptom onset	Approximately 1 month (family recall)	Approximately 1 month (family recall)	19 days	69 days	19 days
Symptoms onset date	23 Feb 2018	3 Jul 2018	13 Sep 2019	29 Aug 2022	27 Sep 2022
Treatment for rabies symptoms	Antimalarials (Coartem, Artesunate), painkillers (Paracetamol, Diclofenac)	Paracetamol	Paracetamol	Normal saline intravenous	Palliative care
Days of illness (symptomatic)	3 days	6 days	4 days	1 day	7 days
Date of death	25 Feb 2018	8 Jul 2018	16 Sep 2019	29 Aug 2022	3 Oct 2022
Diagnostic results	RDT + (frozen brain)	RDT + (frozen brain)	RDT- (frozen brain); IHT + (formalin-fixed brain tissue)	RDT + (fresh brain)	RDT + (fresh brain)
Viral lineages	AF1b_A2	AF1a_A1.1	AF1b_A1.1	AF1a_C1	AF1b_A1.1
Genome coverage (excluding masked sites)	76.75%	51.12%	97.85%	94.24%	97.63%
Accession ID	OR045959	OR045960	OR920212	OR045927	OR045947

All the viruses belong to the Cosmopolitan major clade, and are classified here by minor clade and lineage

*RDT* rapid diagnostic test, *IHT* immunohistochemical test

Case 1 was vaccinated against tetanus, but not advised on rabies post-exposure vaccination despite attending a health facility the same day as being bitten multiple times by an unknown dog, including one bite to the forehead. Twenty-eight days after being bitten, Case 1 started to show rabies symptoms. From symptoms onset the patient was treated for malaria, initially at home, then at a local hospital. The patient’s condition deteriorated rapidly, leading to their transfer to a major referral hospital where they died upon arrival. Although Case 2 had no bite history, the patient had killed his two dogs after they manifested signs of rabies one month prior to his death. After presenting to a nearby health facility with rabies symptoms the patient was transferred to a major referral hospital and pronounced dead 6 days later. Case 3 reported to a local hospital with bites to the lips from an unknown dog, and was vaccinated against rabies following an off-label intramuscular (IM) regimen (1 ml on days 0, 7 and 18). WHO recommendation for IM in use in the country is day 0, 3, 7, 14 and 28. Rabies symptoms began 19 days later, i.e., one day after the third vaccination; and the patient died four days later following transfer to a major referral hospital. Case 4 started post-exposure vaccination, via the intramuscular route, one day after being bitten multiple times on the head and arm by an unknown dog, but did not receive further vaccinations as relatives reported that they were not advised to do so. After symptoms onset (69 days later) the patient was taken back to the health facility where the patient was initially vaccinated, then transferred to a major referral hospital where the patient died shortly thereafter. Case 5 was referred to a major hospital from a health facility where he presented with symptoms of rabies 19 days after exposure. The patient had sought treatment from a traditional healer after being bitten on the leg by his own dog, but otherwise did not receive formal health care after the bite. Palliative care was given until death 7 days after hospital admission. RIG was not administered to any of these patients, despite the site and severity of bites (multiple bites on the forehead and lips) for cases 1, 3 and 4. Further details about each case are as follows:

*Case 1* On 23rd February 2018, a 10-year-old boy from Nyawara village, Gem sub-county, Siaya county, Kenya, presented at a local health facility with fever, headache, and general body weakness. Suspected of having malaria, he was initially treated at home with a single dose of the antimalarial Coartem. His condition worsened the next day, with symptoms including headache, dizziness, restlessness, vomiting, and incoherent speech. At the health facility, a rapid diagnostic test confirmed malaria, and he was given intramuscular Artesunate, with a repeat dose after four hours. The nurse noticed restlessness, aggression to touch, and abnormal vocalisation. Upon inquiry, it was revealed that the boy had been bitten three times on his left forearm and forehead by an unknown dog a month earlier but had not received PEP. The child’s parents reported that on the day of the bite, they had taken him to a local health facility where he received painkillers (Paracetamol), a tetanus vaccination, and wound cleaning with paraffin, but no rabies-related treatment or advice was provided. With evident rabies symptoms, he was referred to the nearest hospital and given more painkillers (Diclofenac). That night, he experienced difficulty swallowing, uncontrollable salivation, and extreme agitation at the sight of liquids. His condition deteriorated further on 25th February, leading to his referral to Siaya County Referral Hospital for palliative care, where he was declared dead on arrival.

*Case 2* On 3rd July 2018, a 37-year-old man from Rarieda village, Gem sub-county, Siaya county, Kenya, visited a local health facility with symptoms of paralysis, abnormal vocalisation, and difficulty breathing. He was given Paracetamol. His condition worsened the following day, and he was transferred to Siaya County Referral Hospital for further treatment. The exact nature of the treatment he received there is unclear. On 8th July 2018, he was pronounced dead due to suspected rabies. Tracing back his exposure history, the family reported no knowledge of any bites from rabid animals. However, it was noted that the man had killed his two dogs after they exhibited signs of rabies, one month and three weeks prior to his own death, respectively. Details on whether the dogs were vaccinated during the mass vaccination campaign conducted in Siaya in 2018 were not disclosed.

*Case 3* On 13th September 2019, a six-year-old girl from Tarakea-Rombo village, Moshi district, Kilimanjaro region, Tanzania, was taken to her local health facility with a headache. She was given painkillers (Paracetamol) and discharged the same day. The next day, her condition worsened with high fever, headache, and hallucinations. She was referred to Huruma district hospital and then to Kilimanjaro Christian Medical Centre (KCMC) referral hospital in Moshi town. On 16th September 2019, 22 days after the bite, she died. Investigation of her exposure history indicated that on 26th August 2019, the girl was bitten multiple times on the upper lip by an unknown dog that ran away after the bite. She reported immediately to her local health facility, received proper wound cleaning, and was administered a first dose of rabies vaccine on day 0 (26th August 2019) via the intramuscular route as well as an anti-tetanus injection. She returned for her second rabies vaccine dose on 2nd September 2019 (day 7), followed by her third dose on 12th September (day 18). The family reported paying 30,000 Tanzania Shillings (Tsh) per vaccination, excluding transportation fees to the health facility, which was 16 km away. Despite receiving these vaccine doses, her symptoms progressed, leading to her death.

*Case 4* On 29th August 2022, a six-year-old boy from Alara village, South West Sakwa ward, Bondo sub-county, Siaya county, Kenya, was taken to a local health facility with complaints of fever, insomnia, abnormal vocalisation, difficulty breathing and swallowing, hallucinations, and restlessness. He was given normal saline intravenous and then transferred to the referral hospital in Bondo, where he received palliative care and died four hours later. According to his exposure history, on 22nd June 2022, the boy was bitten and scratched multiple times on the head and arm by an unknown dog while walking home. The dog was chased away by villagers. The boy received first aid at home, where his wounds were washed with soap and water, and he was then rushed to a traditional herbalist, where he received a concoction of herbs. Upon hearing this news, the community health worker advised the family to take the child to a hospital for PEP. The family took the boy to a local health facility in West Sakwa, Bondo sub-county, where he received the first dose of the rabies vaccine on 23rd June 2022 via the intramuscular route. The family reported paying 1000 Kenya shillings (Ksh) for the vaccine, excluding transportation to the health facility, which was 15 km away. They were given no further advice on the follow-up course of vaccination nor the severity of rabies. Despite receiving the initial vaccine dose, his symptoms progressed, leading to his death.

*Case 5* On 27th September 2022, a thirteen-year-old boy from Bulati village, Ngorongoro district, Arusha region, Tanzania, was admitted to Fame Hospital after being referred from Bulati Health Facility on the same day. The boy exhibited signs of rabies, including excessive salivation, paralysis, abnormal vocalisation, and restlessness. Tracing back his exposure history, the boy had been bitten by his own dog on the left leg on 8th September 2022. Despite regularly attending the hospital for other medical treatments, he did not report the dog bite or receive any treatment from the health facility or hospital. Instead, the boy was brought to a healer where his wound was washed with milk, and a traditional treatment was initiated by placing a coin on the wound to suck out the poison. Upon presenting at the health facility, the medical staff discovered that the boy had been bitten by the dog 19 days prior. He was referred to a major hospital on the same day, where he received palliative care until he died on 3rd October 2022, 25 days after the bite.

**Fig. 1:**
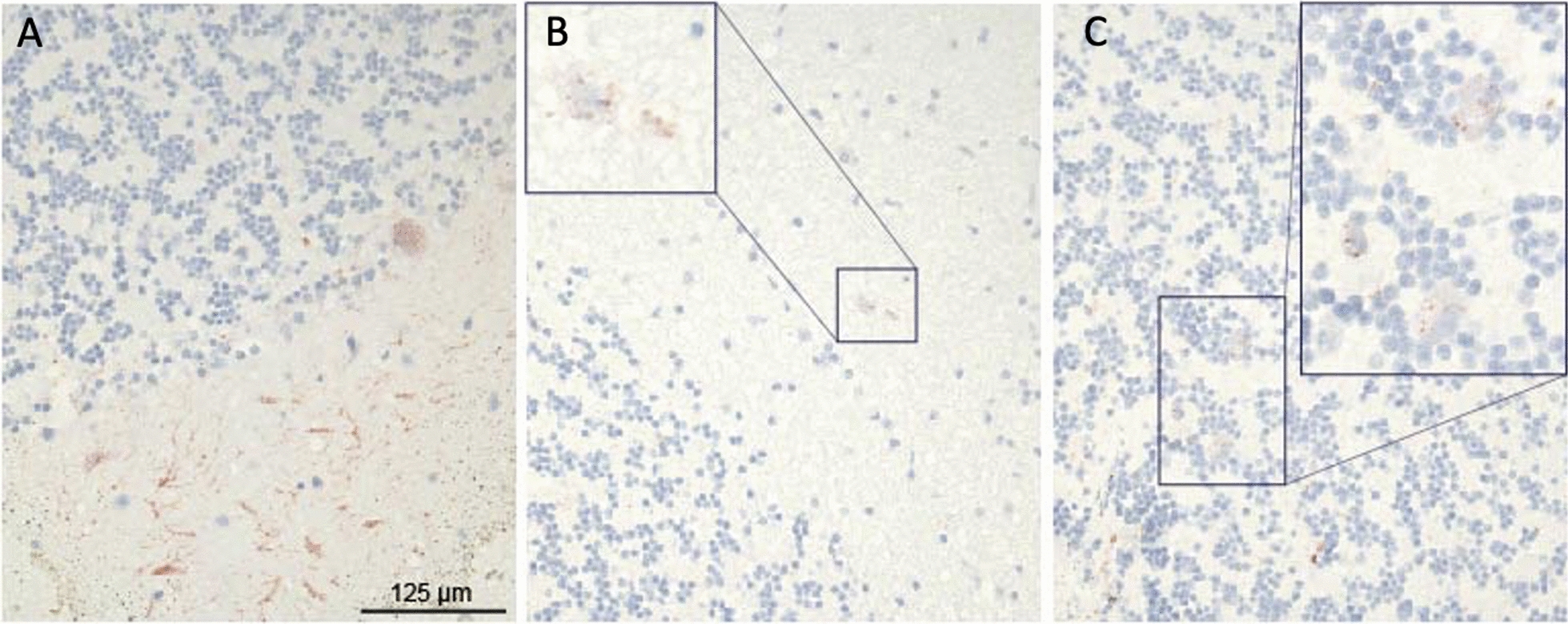
Positive immunohistochemistry staining of frozen brain slides of Case 3. (**A**) Slides at × 40 magnification, and manual zoomed-in of cells of interest **B**) and **C**). The red stain zoomed in, indicates the presence of rabies virus antigen detected with specific antibodies (RABV-N, antibody 5DF12) and streptavidin-biotin complex staining

The rabies incubation period varies; symptoms typically develop days to weeks after infection, but can take months depending on factors such as the bite location and severity [1]. Three of the patients in this case series progressed to rabies within one month of exposure (the date of exposure was not possible to confirm for Case 2, although was recalled to be around one month prior to death), whereas Case 4 developed symptoms more than two months later. Each patient displayed common clinical signs of rabies: fever, abnormal vocalisation, difficulty breathing and swallowing, hallucinations, paralysis, hydrophobia, aggressiveness, excessive salivation and restlessness. All patients except Case 2 had a clear history of a dog bite making the clinical diagnosis straightforward. A history of close contact with two suspect rabid dogs assisted in reaching a diagnosis for Case 2. Samples from four of the five cases were positive by rapid diagnostic test. Case 3 had a negative test result; however, the presence of rabies virus antigen was confirmed by immunohistochemistry, using the streptavidin–biotin complex staining method (Fig. 1).

### Phylogenetic investigation

Amplicon-based sequencing was carried out to compare rabies viruses from the five human cases to those from recent animal rabies cases in the region using a previously described protocol [23]. Full details of the laboratory procedures are found in Supplementary File 1. Due to the poor sample conditions and primer mismatches related to the early primer set used (i.e., targeting rabies virus diversity in Tanzania from 2019–2020 but not optimised for samples from Kenya), a few of the sequences generated (14/98) had less than 90% genome coverage. Sequences from Cases 1 and 2 from Kenya both had less than 90% genome coverage (Supplementary Table 1).

**Fig. 2:**
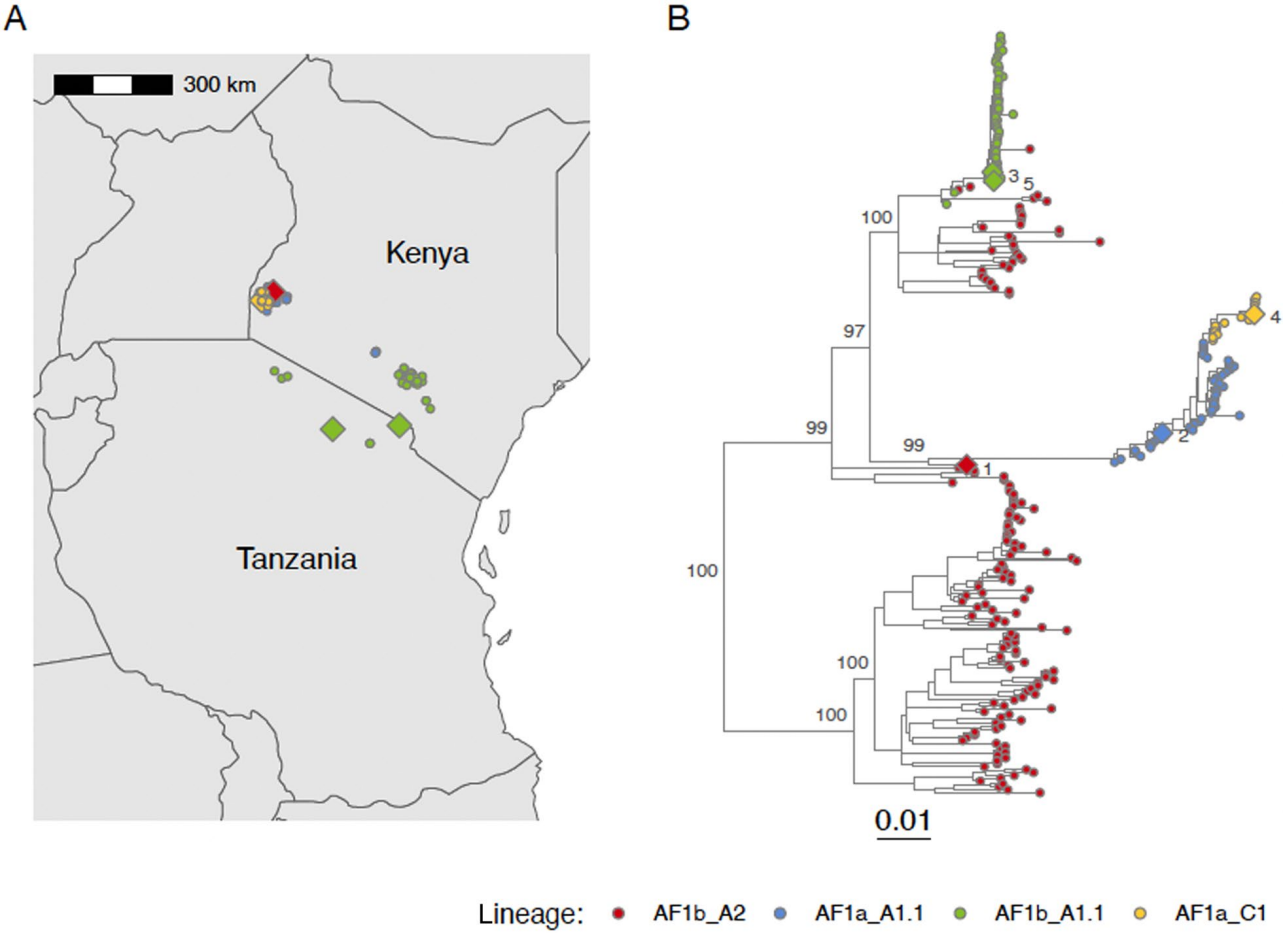
Rabies viruses from five human rabies cases and animal rabies cases from the same viral lineages. **A** Sequenced cases in East Africa and **B** maximum likelihood tree of sequences (*n* = 262). Sequences from the Arctic AL1a clade (GenBank accession AB699220, AY956319, EF437215, HE802675, HE802676, KF154996, KY775603, KY775604, LT909539, LT909541 and MG099711) were used as an outgroup (not shown) to root the tree. Tips and points are coloured by lineage, with diamonds and numbers denoting the human cases (Case 1 sequence OR045959 from 27 Feb 2018, Case 2 sequence OR045960 from 18 Jul 2018, Case 3 sequence OR920212 from 16 Sep 2019, Case 4 sequence OR045927 from 29 Aug 2022, and Case 5 sequence OR045947 from 3 Oct 2022) and circles denoting animal cases. Scale in substitutions/site. Ultrafast bootstrap values of lineage-defining nodes shown

All the sequenced viruses were from the Cosmopolitan clade; Cases 2 and 4 belonged to minor clade AF1a (both from Kenya), and Cases 1, 3 and 5 belonged to minor clade AF1b (from Kenya, Tanzania and Tanzania respectively) (Fig. 2). Cases 1, 2, 3 and 5 were from previously reported circulating lineages, with Cases 3 and 5 from the same lineage (AF1b_A1.1), while Case 4 was from a newly designated lineage (AF1a_C1). The most closely related antecedent and subsequent sequences to all the human cases were from domestic dogs, except for the subsequent sequence to Case 2 which was from a cow, indicating likely spill over from the lineage which was circulating in domestic dogs.

**Fig. 3:**
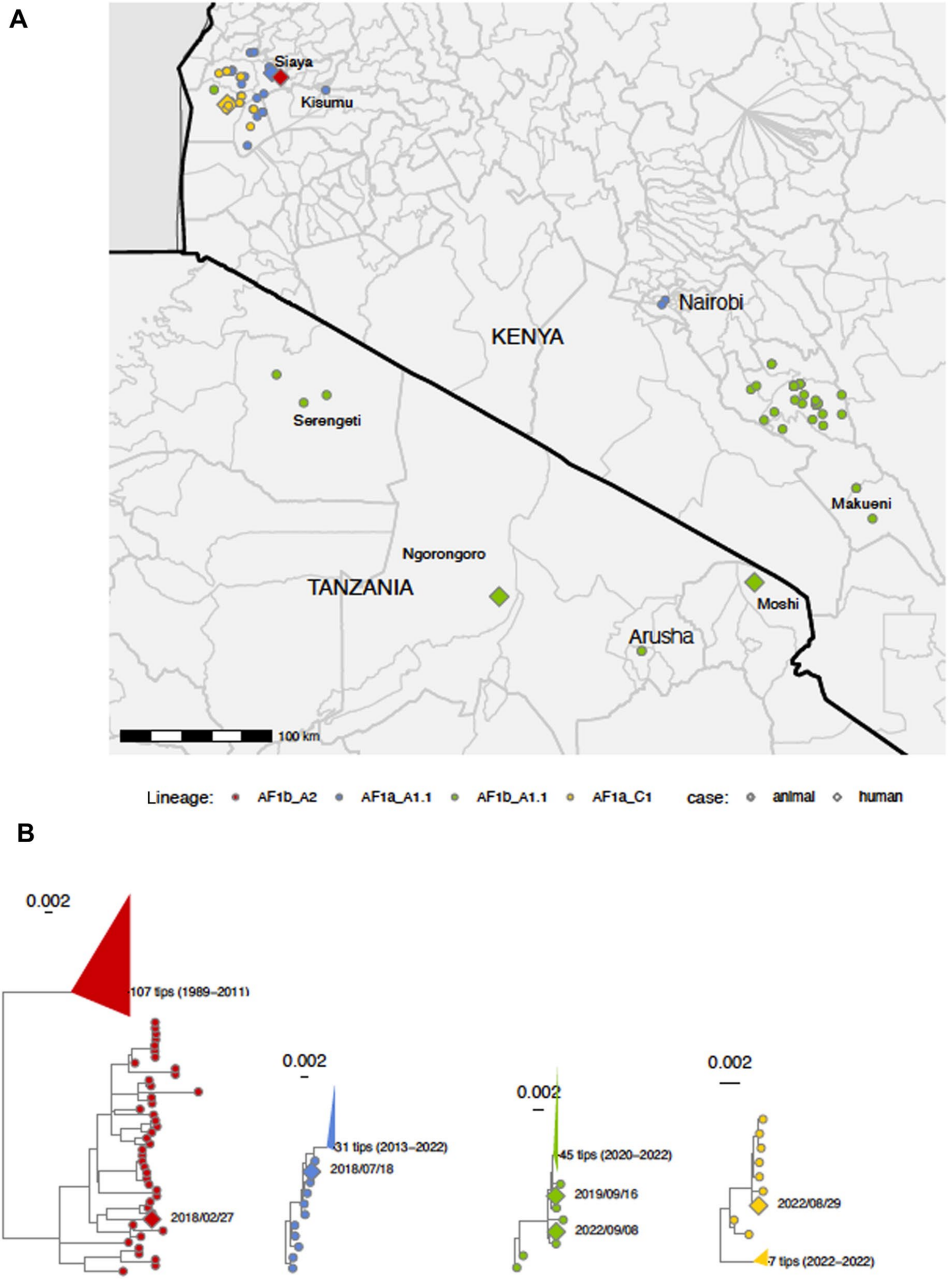
Geolocations of RABV sequences and subtrees of human and animal cases in Kenya and Tanzania. **A** The map shows the locations of the sequenced human and animal cases from East Africa coloured by lineage. **B** Phylogenetic subtrees shown for lineage AF1b_A2 (*n* = 151, Case 1) in red, for lineage AF1a_A1.1 (*n* = 42, Case 2) in blue, for lineage AF1b_A1.1 (*n* = 53, Cases 3 and 5) in green and for lineage AF1a_C1 (*n* = 16, Case 5) in yellow. Human cases denoted by diamonds (OR045959, OR045960, OR920212, OR045947, and OR045927). Relevant locations labelled. Scale in substitutions/site, and outgroup rooted with ordered nodes

All cases except Case 4 represent cross-border lineages, with lineage AF1b_A2, widespread across Africa but only reported from East Africa in 2018 with this human death (Case 1) in Kenya (Fig. 3). The most closely related antecedent sequence to Case 1 is from Bangui, CAR, where over 85% of cases in this lineage were also from (Fig. 2A). The geographic distance and phylogenetic divergence between these cases indicate limited wider sampling of the lineage, which likely originated decades ago (supplementary Table 2) and is now widespread, though largely undetected, across Africa. In contrast, lineage AF1b_A1.1 (Cases 3 and 5) has been seen exclusively in East Africa; first seen in Uganda in 2009, then Tanzania in 2011 followed by Kenya in 2020, where it has been repeatedly detected in Makueni county. Conversely, detection in Tanzania, has been sporadic and near the Kenyan border (Fig. 3A), suggestive of cross-border spread. The closest antecedent sequence is the same for both Cases 3 and 5—a rabid dog from Serengeti District in Tanzania sampled in 2019 (Fig. 3A). Lineage AF1a_A1.1 (Case 2), was originally detected in Ethiopia in 1987, then in Morocco in 1989 where it was seen frequently until 2008. There have also been infrequent detections of AF1a_A1.1 in Algeria since 2000. Virus infections from this lineage were first detected in Kenya in 2013 with human cases in both Nairobi and Siaya (Fig. 3B). Lineage AF1a_C1 (Case 4) is newly designated and highly localised, found exclusively in Siaya County, Kenya since 2021. The detection of three lineages (corresponding to Cases 1, 2 and 4) all within years or months of each other within Siaya County (Fig. 3) highlight the apparently localised co-circulation of lineages.

## Discussion and conclusions

Through this case series, we highlight critical steps needed to combat the problem of rabies in East Africa. Human rabies deaths occur when rabies-exposed victims fail to receive timely or appropriate PEP. Medical practitioners urgently need training about the risk of rabies and on effective bite patient management [24]. The high cost of PEP remains the most immediate obstacle for rabies-exposed patients [10], compounded by structural factors leading to poor supply and shortages in East Africa. Gavi’s investment in human rabies vaccines should be used to overcome these challenges. Unfortunately, human rabies cases are also still rarely diagnosed. To improve the rate at which cases are diagnosed, we suggest rapid tests can be carefully deployed to confirm human cases, given the absence of decentralised laboratory capacity and highly trained personnel. Our report demonstrates the feasibility of improving human rabies surveillance in East Africa (all 5 cases were identified via surveillance networks initiated by research projects), but also only begins to reveal the scale of the rabies burden. Most human rabies deaths within these communities are not confirmed, while the rabies virus sequences indicate that circulating viral lineages remain largely uncharacterized.

This case series reveals the multiple challenges that bite victims face in obtaining PEP, including inappropriate advice from medical staff not fully aware of the dangers of rabies (Case 1, 4 and 5) or not trained in correct PEP administration (Cases 3 and 4); high costs that act as obstacles to initiating (Case 1) and completing PEP courses (Case 4) as well as more general lack of understanding about rabies risks. This study reports how traditional yet ineffective healing practices are still commonplace (Cases 4 and 5), with two cases (Cases 2 and 5) only visiting hospitals after symptoms onset, when death is inevitable. Human rabies deaths in East Africa are typically not confirmed or reported and as a result rabies receives negligible investment for prevention and control. The clinical history of these five human cases together with use of rapid diagnostic tests and immunohistochemistry enabled their confirmation, while sequencing provided further epidemiological context as to their source of origin.

### Improving rabies diagnosis and surveillance

Human rabies diagnosis remains a challenge in low- and middle-income countries (LMICs) and cases are often misdiagnosed, for example, Case 1 was considered malaria. If a history of rabies exposure is elicited, as for all five cases reported here (although atypical for Case 2), clinical presentation of furious rabies is diagnostic, but paralytic rabies can be more difficult to identify [6]. Ante-mortem diagnostic tests include antigen detection, antibody assays and virus isolation, but all have limited success [9]. Post-mortem tests are rarely performed due to lack of personnel trained to collect samples, lack of accredited laboratories (Biosafety Level 2 or 3) for diagnosis and because many clinical cases return home in the absence of palliative care options and are subsequently not reported within surveillance networks which might allow the possibility of sample collection [17]. The direct fluorescent antibody test (DFA) is the recommended “gold standard” for post-mortem diagnosis [25], but requires fluorescence microscopy which is expensive and limited in availability. The Direct Rapid Immunohistochemical Test which has similar sensitivity and specificity to the DFA and only requires light microscopy [26] is based on a simplified version of the immunohistochemistry diagnostic assay [27]. Immunohistochemistry was performed on one of the five cases reported here (Case 3) at an overseas laboratory (Netherlands) through ongoing research, but is not carried out in East Africa. Rapid diagnostic tests show promise, being successfully used here to diagnose 4 cases in situ. The negative result for Case 3 was likely due to sample storage (in formalin). More generally freeze–thaw cycles compromise the sensitivity of these tests which are recommended for use only on fresh brain samples. Though rapid diagnostic tests may increase human rabies diagnosis, they are not yet recommended by international organisations. Concerns remain about test sensitivity and quality control [25, 27], particularly with respect to PEP decision-making. Laboratory comparisons of rapid test brands under different protocols have been inconsistent, with batch variability presenting an issue [28, 29]. Nonetheless, the best performing test has been found to have high sensitivity on fresh samples [30, 31]. In our situation, we found rapid tests provided a valuable diagnostic that was possible to quickly and easily perform in the absence of alternatives, and where the risk of rabies was already apparent.

Molecular techniques for diagnosis such as PCR and sequencing are promising and further help in understanding rabies virus biology, molecular epidemiology, pathogenesis and sources of transmission [13]*.* The viral genomic data associated with the cases presented here highlights the role of domestic dogs in maintaining rabies circulation and resultant risk to humans. These deaths could be avoided if the disease was eliminated from source populations of domestic dogs through mass vaccination, which remains the most cost-effective measure for rabies prevention in endemic regions. Furthermore, the genomic data uncover population connectivity and frequent transboundary viral spread (Cases 1, 3 and 5) indicating the need for regional planning and coordinated dog vaccination, as well as for much improved surveillance. Further interpretation is limited by the availability of sequences, though lineage assignment begins to reveal the extent to which under sampling is a problem. Our recent sequencing identified the lineage AF1b_A2 for the first time in Kenya, and a new lineage, AF1a_C1 only seen in Kenya. The new lineage designation and considerable divergence of the most closely related sequences to Case 1 within lineage AF1b_A2 (Supplementary Table 1) illustrate the negligible sampling of circulating rabies viruses.

### Improving access to post-exposure prophylaxis

WHO now recommends an intradermal post-exposure vaccination regimen that can be completed in one week, requiring visits only on day 0, day 3 and day 7 respectively [1]. This highly effective abridged regimen is both dose-sparing and more economical for patients and health providers [32]. Yet, rabies endemic countries have been slow to adopt the updated WHO position. In parts of Tanzania a post-exposure vaccination regimen is used that is not recommended (Case 3). Moreover, while advised for WHO category III exposures such as Cases 1, 3 and 4, RIG has rarely been available in East Africa. Concern has been raised regarding recent human deaths in Tanzania attributed to confusion in post-exposure management, with RIG speculated to have been given and not vaccination.

Potential barriers to accessing PEP include lack of awareness among healthcare providers and the public, and economic decisions that prevent the vaccine being stocked or made available free-of-charge to patients. Many healthcare facilities in endemic regions are under-resourced and healthcare practitioners may not be adequately trained to administer PEP correctly, leading to inconsistent practices and poor patient outcomes. To address these barriers, there is an urgent need to update national guidelines to follow WHO guidance and ensure healthcare practitioners are trained and competent to manage rabies exposures appropriately. WHO has developed new policies and guidance for countries [1, 33], and Gavi’s support for human rabies vaccines provides an opportunity to update national guidelines, and operationalize these through vaccine procurement and distribution, with delivery of training packages for healthcare practitioners and integration of guidance into health curricula. Community education campaigns are also crucial to raise awareness about the importance of timely PEP and proper wound care after an exposure.

The high cost of vaccines remains a barrier for bite victims (typically costing around USD 10 per vaccination in East Africa, for example, Case 3 paid 30,000 Tanzania shillings per dose, equivalent to USD 13 while Case 4 paid 1000 Kenya shillings per dose, equivalent to USD 8) as well as for health providers, translating into inadequate supply and chronic stockouts. Meanwhile, indirect costs to patients (travel, lost income) also can be prohibitively high. The promise of investment in human rabies vaccines by Gavi, the Vaccine Alliance, offers a chance to address PEP access issues and radically redress inequalities underlying human rabies deaths [8, 24]. Estimates from modelling studies suggest improved PEP access would prevent over 1.3 million human rabies deaths by 2035 [2]. But with global health priorities disrupted by the pandemic, Gavi support for human rabies vaccines has yet to begin and these deaths continue.

### Recommendations to address rabies from a One Health perspective

Thousands of people every year in East Africa still face challenges in accessing life-saving PEP. If Gavi delivers on its proposed investment, it can address the market failure in access to lifesaving rabies vaccines [2], catalysing progress on this One Health pathway. We present the first whole genome sequences from human rabies cases generated in East Africa. Viral genomic data support the role of domestic dogs in maintaining rabies circulation in the region and the resultant risk to humans. Research across large parts of Tanzania demonstrates that domestic dogs maintain rabies virus circulation [34–36], in settings with abundant wildlife and even where wildlife cases are common [37]. However, misperceptions of wildlife being responsible for rabies persistence, still act as a barrier to implementing rabies control in domestic dog populations [38]. A One Health approach is necessary to reduce the burden of rabies, comprising the scaling up of mass dog vaccination to interrupt transmission in reservoir populations, improving access to PEP while rabies continues to circulate, and public education to ensure participation in dog vaccination campaigns and improved health-seeking for PEP. As countries pursue the global ‘Zero by 30’ goal to eliminate human deaths from dog-mediated rabies [38], coordinated cross-border dog vaccination programs must be emphasised as a long-term solution to rabies control. Genomic approaches have the potential to enhance rabies surveillance and provide actionable information, for example by revealing transboundary spread. We urge regional coordinated action towards this goal to prevent these tragic deaths and recommend that future research and policy focus on comprehensive dog vaccination to control rabies and enhanced surveillance to evaluate progress.

## Supplementary Information

Additional file 1

Additional file 2

Additional file 3

## Data Availability

Data and code to reproduce the analyses and figures are available from our public repository https://github.com/Gurdeepjaswant/EA_human_rabies_case_series. 10.5281/zenodo.13934433.

## References

[1] World Health Organisation. Rabies vaccine: WHO position paper. Wkly Epidemiol Rec. 2018;16:201–20.

[2] Hampson K, Ventura F, Steenson R, Mancy R, Trotter C, Cooper L, et al. The potential effect of improved provision of rabies post-exposure prophylaxis in Gavi-eligible countries: a modelling study. Lancet Infect Dis. 2019;19:102–11. 10.1016/S1473-3099(18)30512-7. 30472178 10.1016/S1473-3099(18)30512-7 PMC6300480

[3] Thiptara A, Atwill ER, Kongkaew W, Chomel BB. Epidemiologic trends of rabies in domestic animals in southern Thailand, 1994–2008. Am J Trop Med Hyg. 2011;85:138–45. 10.4269/ajtmh.2011.10-0535. 21734139 10.4269/ajtmh.2011.10-0535 PMC3122358

[4] Nel LH. Discrepancies in data reporting for rabies. Africa Emerg Infect Dis. 2013;19:529–33. 10.3201/eid1904.120185. 23628197 10.3201/eid1904.120185 PMC3647406

[5] Fooks AR, Banyard AC, Horton DL, Johnson N, McElhinney LM, Jackson AC. Current status of rabies and prospects for elimination. Lancet. 2014;384:1389–99. 10.1016/S0140-6736(13)62707-5. 24828901 10.1016/S0140-6736(13)62707-5 PMC7159301

[6] Rasooli A, Pourhossein B, Bashar R, Shirzadi MR, Amiri B, Kheiri EV, et al. Investigating possible etiologies of post-exposure prophylaxis failure and deaths from rabies infection: case reports. Int J Med Toxicol Forens Med. 2020;10:27378–27378. 10.32598/ijmtfm.v10i3.27378.

[7] World Health Organisation. WHO Expert Consultation on Rabies.World Health Organization technical report series. 2018. https://www.who.int/publications/i/item/WHO-TRS-1012.

[8] Thumbi SM, Blumberg L, le Roux K, Salahuddin N, Abela B. A call to accelerate an end to human rabies deaths. Lancet. 2023;400:2261–4. 10.1016/S0140-6736(22)02487-4. 10.1016/S0140-6736(22)02487-4 PMC9754655 36528379

[9] Warrell MJ, Warrell DA. Rabies and other lyssavirus diseases. Lancet. 2004;363:959–69. 10.1016/S0140-6736(04)15792-9. 15043965 10.1016/S0140-6736(04)15792-9

[10] Changalucha J, Steenson R, Grieve E, Cleaveland S, Lembo T, Lushasi K, et al. The need to improve access to rabies post-exposure vaccines: lessons from Tanzania. Vaccine. 2019;37:A45–53. 10.1016/j.vaccine.2018.08.086. 30309746 10.1016/j.vaccine.2018.08.086 PMC6863039

[11] Soun VV, Eidson M, Wallace BJ, Drabkin PD, Jones G, Leach R, et al. Antemortem diagnosis of New York human rabies case and review of U.S. Cases. Int J Biomed Sci. 2006;2:434–45. 23675013 PMC3614649

[12] Madhusudana SN, Sukumaran SM. Antemortem diagnosis and prevention of human rabies. Ann Indian Acad Neurol. 2008;11:3–12. 10.4103/0972-2327.40219. 19966972 10.4103/0972-2327.40219 PMC2781142

[13] Talbi C, Lemey P, Suchard MA, Abdelatif E, Elharrak M, Jalal N, et al. Phylodynamics and human-mediated dispersal of a zoonotic virus. PLOS Pathog. 2010;6: e1001166. 10.1371/journal.ppat.1001166. 21060816 10.1371/journal.ppat.1001166 PMC2965766

[14] Trewby H, Nadin-Davis SA, Real LA, Biek R. Processes underlying rabies virus incursions across US–Canada Border as revealed by whole-genome phylogeography. Emerg Infect Dis. 2017;23:1454–61. 10.3201/eid2309.170325. 28820138 10.3201/eid2309.170325 PMC5572885

[15] Lushasi K, Brunker K, Rajeev M, Ferguson EA, Jaswant G, Baker LL, et al. Integrating contact tracing and whole-genome sequencing to track the elimination of dog-mediated rabies: an observational and genomic study. Elife. 2023;12: e85262. 10.7554/eLife.85262. 37227428 10.7554/eLife.85262 PMC10299823

[16] Layan M, Dellicour S, Baele G, Cauchemez S, Bourhy H. Mathematical modelling and phylodynamics for the study of dog rabies dynamics and control: a scoping review. PLoS Negl Trop Dis. 2021;15: e0009449. 10.1371/journal.pntd.0009449. 34043640 10.1371/journal.pntd.0009449 PMC8189497

[17] McElhinney LM, Marston DA, Golding M, Nadin-Davis SA. Chapter 12 - Laboratory diagnosis of rabies. In: Fooks AR, Jackson AC, editors. rabies. 4th ed. Boston: Academic Press; 2020. p. 401–44.

[18] Mollentze N, Weyer J, Markotter W, le Roux K, Nel LH. Dog rabies in southern Africa: regional surveillance and phylogeographical analyses are an important component of control and elimination strategies. Virus Genes. 2013;47:569–73. 10.1007/s11262-013-0974-3. 23996607 10.1007/s11262-013-0974-3

[19] Coetzee P, Weyer J, Paweska JT, Burt FJ, Markotter W, Nel LH. Use of a molecular epidemiological database to track human rabies case histories in South Africa. Epidemiol Infect. 2008;136:1270–6. 10.1017/S0950268807009582. 17961278 10.1017/S0950268807009582 PMC2870926

[20] Faye M, Faye O, Paola ND, Ndione MHD, Diagne MM, Diagne CT, et al. Rabies surveillance in Senegal 2001 to 2015 uncovers first infection of a honey-badger. Transbound Emerg Dis. 2022;69:e1350–64. 10.1111/tbed.14465. 35124899 10.1111/tbed.14465

[21] Ogo MF, Nel LH, Sabeta CT. Phylogenetic evidence of the public and veterinary health threat of dog rabies in Nigeria. Nig Vet J. 2011. 10.4314/nvj.v32i1.68996.

[22] Hampson K, Dobson A, Kaare M, Dushoff J, Magoto M, Sindoya E, et al. Rabies exposures, post-exposure prophylaxis and deaths in a region of endemic canine rabies. PLoS Negl Trop Dis. 2008;2: e339. 10.1371/journal.pntd.0000339. 19030223 10.1371/journal.pntd.0000339 PMC2582685

[23] Bautista C, Jaswant G, French H, Campbell K, Durrant R, Gifford R, et al. Whole genome sequencing for rapid characterization of rabies virus using nanopore technology. J Vis Exp. 2023. 10.3791/65414. 37677046 10.3791/65414

[24] Wentworth D, Hampson K, Thumbi SM, Mwatondo A, Wambura G, Rui N. A social justice perspective on access to human rabies vaccines. Vaccine. 2019. 10.1016/j.vaccine.2019.01.065. 30952501 10.1016/j.vaccine.2019.01.065 PMC7612387

[25] Rupprecht CE, Fooks AR, Abela-Ridder B. Laboratory techniques in rabies. 5th ed. World Health Organization: Geneva; 2019.

[26] Lembo T, Niezgoda M, Velasco-Villa A, Cleaveland S, Ernest E, Rupprecht CE. Evaluation of a direct, rapid immunohistochemical test for rabies diagnosis. Emerg Infect Dis. 2006;12:310–3. 10.3201/eid1202.050812. 16494761 10.3201/eid1202.050812 PMC3294322

[27] Coetzer A, Nel LH, Taylor L. Direct, Rapid Immunohistochemical Test (DRIT). Global Alliance for Rabies Control; 2017. https://rabiesalliance.org/resource/direct-rapid-immunohistochemistry-test-drit-manual. Accessed 20 Jun 2024.

[28] Klein A, Fahrion A, Finke S, Eyngor M, Novak S, Yakobson B, et al. Further evidence of inadequate quality in lateral flow devices commercially offered for the diagnosis of rabies. Trop Med Infect Dis. 2020;5:13. 10.3390/tropicalmed5010013. 31963635 10.3390/tropicalmed5010013 PMC7157750

[29] Eggerbauer E, de Benedictis P, Hoffmann B, Mettenleiter TC, Schlottau K, Ngoepe EC, et al. Evaluation of six commercially available rapid immunochromatographic tests for the diagnosis of rabies in brain material. PLoS Negl Trop Dis. 2016;10: e0004776. 10.1371/journal.pntd.0004776. 27336943 10.1371/journal.pntd.0004776 PMC4918935

[30] Mauti S, Léchenne M, Naïssengar S, Traoré A, Kallo V, Kouakou C, et al. Field postmortem rabies rapid immunochromatographic diagnostic test for resource-limited settings with further molecular applications. J Vis Exp. 2020. 10.3791/60008. 32658185 10.3791/60008

[31] Léchenne M, Naïssengar K, Lepelletier A, Alfaroukh IO, Bourhy H, Zinsstag J, et al. Validation of a rapid rabies diagnostic tool for field surveillance in developing countries. PLoS Negl Trop Dis. 2016;10: e0005010. 10.1371/journal.pntd.0005010. 27706156 10.1371/journal.pntd.0005010 PMC5051951

[32] Cantaert T, Borand L, Kergoat L, Leng C, Ung S, In S, et al. A 1-week intradermal dose-sparing regimen for rabies post-exposure prophylaxis (RESIST-2): an observational cohort study. Lancet Infect Dis. 2019;19:1355–62. 10.1016/S1473-3099(19)30311-1. 31570311 10.1016/S1473-3099(19)30311-1

[33] Nadal D, Bote K, Masthi R, Narayana A, Ross Y, Wallace R, et al. Rabies post-exposure prophylaxis delivery to ensure treatment efficacy and increase compliance. JID One Health. 2023;1: 100006. 10.1016/j.ijidoh.2023.100006. 10.1016/j.ijidoh.2023.100006 PMC10752235 38152594

[34] Mancy R, Rajeev M, Lugelo A, Brunker K, Cleaveland S, Ferguson EA, et al. Rabies shows how scale of transmission can enable acute infections to persist at low prevalence. Science. 2022;376:512–6. 10.1126/science.abn0713. 35482879 10.1126/science.abn0713 PMC7613728

[35] Hampson K, Dushoff J, Cleaveland S, Haydon DT, Kaare M, Packer C, et al. Transmission dynamics and prospects for the elimination of canine Rabies. PLoS Biol. 2009;7:0462–71. 10.1371/journal.pbio.1000053. 10.1371/journal.pbio.1000053 PMC2653555 19278295

[36] Lushasi K, Hayes S, Ferguson EA, Changalucha J, Cleaveland S, Govella NJ, et al. Reservoir dynamics of rabies in south-east Tanzania and the roles of cross-species transmission and domestic dog vaccination. J Appl Ecol. 2021;58:2673–85. 10.1111/1365-2664.13983. 35221371 10.1111/1365-2664.13983 PMC7612421

[37] Lembo T, Hampson K, Kaare MT, Ernest E, Knobel D, Kazwala RR, et al. The feasibility of canine rabies elimination in africa: dispelling doubts with data. PLoS Negl Trop Dis. 2010;4: e626. 10.1371/journal.pntd.0000626. 20186330 10.1371/journal.pntd.0000626 PMC2826407

[38] Minghui R, Stone M, Semedo MH, Nel L. New global strategic plan to eliminate dog-mediated rabies by 2030. Lancet Global Health. 2018;6:e828–9. 10.1016/S2214-109X(18)30302-4. 29929890 10.1016/S2214-109X(18)30302-4

