# Supplementary material for: Molecular characterisation of human rabies in Tanzania and Kenya: a case series report and phylogenetic investigation
Source: Infect Dis Poverty. 2024 Oct 28;13:79. doi: 10.1186/s40249-024-01245-w (PMC11514914; doi:10.1186/s40249-024-01245-w)
Supplement: Supplementary file 1 — Additional file 1 [file 40249_2024_1245_MOESM1_ESM.docx]

**Supplementary File 1: Rabies diagnosis and sequencing**

*Diagnosis*

Post-mortem brain samples were collected by pathologists at the hospitals where the deceased attended. Samples from case 1 were collected two days after death whereas samples from all other cases were collected the same day.  Testing and sequencing was conducted at the Kilimanjaro Clinical Research Institute (KCRI), Tanzania, and at the University of Nairobi Institute of Tropical and Infectious Diseases (UNITID), Kenya. Samples from cases 1 and 2 were stored for over a year at UNITID at -80°C before testing, while samples from case 3 were stored for one month at KCRI at -80°C. Samples from cases 4 and 5 were tested on the same day as collection, then stored for varying periods at -80°C before sequencing. All samples were tested with rapid diagnostic tests (Antigen Rapid Rabies Ag Test Kit, BioNote) following the manufacturer’s instructions [1]. Additionally, a brain specimen from case 3 was fixed in formalin for immunohistochemistry [2] at the Department of Viroscience, Rotterdam, the Netherlands. The sample was first embedded in paraffin, cut in 3µM slices, stained with antibody against the nucleoprotein (RABV-N, antibody 5DF12) then with peroxidase-labelled goat-anti-mouse IgG1. Peroxidase was revealed with aminoethyl carbazole, resulting in a red precipitate. Slides were counterstained with hematoxylin. Isotype controls (Streptavidin-biotin complex) were included to assess specific staining [3].

*Sequencing*

Brain samples were prepared and sequenced following a previously established end-to-end protocol [4]. In brief, brain tissue was homogenised and inactivated in RNA shield (Zymo Research, USA). Total RNA was extracted using the Quick Viral RNA Mini-prep Kit (Zymo Research, USA), followed by cDNA synthesis with LunaScript RT Supermix (New England Biolabs, UK). To enrich the RABV DNA, multiplex PCR was conducted following the widely used “ARTIC-method” [5,6], using previously designed primers specific to RABV in East Africa [7], in a reaction with Q5 Hot start High Fidelity 2x Master Mix (New England Biolabs), nuclease-free water and cDNA. Sequencing was undertaken using a Nanopore MinION MK1B, with a ligation-based library preparation kit (SQK-LSK109) and native barcoding (NBD-104 to 196). The final library was loaded on an R9.4.1 flow cell. A negative control of nuclease-free water was included in every run for quality control.  In total, four runs were done for the five cases; the first run included cases 1 and 2, and the remaining three runs were for cases 3, 4, and 5. Other samples from different hosts (domestic dogs and various livestock)  were included in each run, generating 99 new sequences (Supplementary Table 1).

*Bioinformatics*

A previously published bioinformatics pipeline [6] was applied to generate a consensus sequence from raw MinION reads. This included basecalling and barcode demultiplexing using MinKNOW or stand-alone Guppy software (Version 6.3.2). Reads were mapped to a RABV reference sequence from East Africa (GenBank accession number KF155002) using Minimap followed by variant calling and generation of a consensus sequence using Medaka [8]. Genome coverage was calculated using 20 read depth coverage (Supplementary Table 2). Consensus FASTA files are available on NCBI GenBank (accession numbers OR045927 to OR920351).

*Phylogenetics*

RABV-GLUE was used for major and minor phylogenetic clade assignment [9], followed by more resolved lineage designation and assignment using MADDOG [10]. Sequenced viruses were classified using the  nomenclature <Major clade Minor clade_Lineage>, e.g. Cosmopolitan AF1a_A1.1. To build contextual datasets, publicly available sequences from the identified lineages were obtained from RABV-GLUE, and aligned with the newly generated sequences using default settings in MAFFT [11]. Maximum likelihood trees were built using IQTREE2 with model selection [12] and 1000 ultrafast bootstrap replicates [13]. Trees of widespread lineages were checked to identify other closely related cases outside of East Africa relevant to human cases. If absent, these lineages were subset to only include sequences from Kenya or Tanzania up to 10 years prior to each human case (i.e. only sequences relevant to understanding transmission to the human case). Patristic distances (phylogenetic distance between tip pairs) were calculated using the R package castor [14]. Locations of cases were mapped using precise coordinates where available. Where exact coordinates were not available, the village or county centroid was used and jittered for mapping. Cases with no within-country location information were excluded from maps but included in phylogenetic analyses.

Reference

1. Rabies Ag - BioNote - Canine Rapid Test - Pet Rapid Test. [cited 20 Oct 2022]. Available: https://www.qbiotech.gr/pet-rapid-test/canine-rapid-test/bionote-canine/rabies-ag.html?___store=en&___from_store=el

2. Niezgoda M, Satheshkumar PS. Immunohistochemistry Test for the Lyssavirus Antigen Detection from Formalin-Fixed Tissues. JoVE J Vis Exp. 2021; e60138. doi:10.3791/60138

3. CDC - Diagnosis: Immunohistochemistry (IHC) - Rabies. 10 May 2022 [cited 20 Oct 2022]. Available: https://www.cdc.gov/rabies/diagnosis/immunohistochemistry.html

4. Bautista C, Jaswant G, French H, Campbell K, Durrant R, Gifford R, et al. Whole Genome Sequencing for Rapid Characterization of Rabies Virus Using Nanopore Technology. J Vis Exp JoVE. 2023. doi:10.3791/65414

5. Quick J, Grubaugh ND, Pullan ST, Claro IM, Smith AD, Gangavarapu K, et al. Multiplex PCR method for MinION and Illumina sequencing of Zika and other virus genomes directly from clinical samples. Nat Protoc. 2017;12: 1261–1266. doi:10.1038/nprot.2017.066

6. Brunker K, Jaswant G, Thumbi SM, Lushasi K, Lugelo A, Czupryna AM, et al. Rapid in-country sequencing of whole virus genomes to inform rabies elimination programmes. Wellcome Open Res. 2020;5: 3. doi:10.12688/wellcomeopenres.15518.2

7. PrimalScheme: primer panels for multiplex PCR. [cited 20 Oct 2022]. Available: https://primalscheme.com/

8. Medaka. Oxford Nanopore Technologies; 2022. Available: https://github.com/nanoporetech/medaka

9. Singer JB, Thomson EC, McLauchlan J, Hughes J, Gifford RJ. GLUE: a flexible software system for virus sequence data. BMC Bioinformatics. 2018;19: 532. doi:10.1186/s12859-018-2459-9

10. Campbell K, Gifford RJ, Singer J, Hill V, O’Toole A, Rambaut A, et al. Making genomic surveillance deliver: A lineage classification and nomenclature system to inform rabies elimination. PLOS Pathog. 2022;18: e1010023. doi:10.1371/journal.ppat.1010023

11. Katoh K, Misawa K, Kuma K, Miyata T. MAFFT: a novel method for rapid multiple sequence alignment based on fast Fourier transform. Nucleic Acids Res. 2002;30: 3059–3066. doi:10.1093/nar/gkf436

12. Minh BQ, Schmidt HA, Chernomor O, Schrempf D, Woodhams MD, von Haeseler A, et al. IQ-TREE 2: New Models and Efficient Methods for Phylogenetic Inference in the Genomic Era. Mol Biol Evol. 2020;37: 1530–1534. doi:10.1093/molbev/msaa015

13. Minh BQ, Nguyen MAT, von Haeseler A. Ultrafast Approximation for Phylogenetic Bootstrap. Mol Biol Evol. 2013;30: 1188–1195. doi:10.1093/molbev/mst024

14. Using the Castor API R package for data analysis. In: Castor [Internet]. [cited 2 May 2023]. Available: https://helpdesk.castoredc.com/link-castor-to-another-application/using-the-castor-api-r-package-for-data-analysis
