# Supplementary material for: Molecular characterisation of human rabies in Tanzania and Kenya: a case series report and phylogenetic investigation
Source: Infect Dis Poverty. 2024 Oct 28;13:79. doi: 10.1186/s40249-024-01245-w (PMC11514914; doi:10.1186/s40249-024-01245-w)
Supplement: Supplementary file 3 — Additional file 3 [file 40249_2024_1245_MOESM3_ESM.docx]

**Supplementary Table 2. Estimates of divergence between each human case and the most closely related sequenced animal cases.** The minimal divergence was determined by the lowest patristic distance between the human case and related cases from the same lineage. Patristic distance (substitutions per site) and time between each human case and the named related sequence extracted from the phylogeny. The patristic distance was divided by the average mutation rate (0.000244 substitutions per site per year [1]) and used to estimate the time since the cases diverged (equivalent to an approximate most recent common ancestor). - or + indicates days before or after human cases respectively. For cases 1, 3 and 5, no cases from the same lineage have been sequenced since the human case so only the nearest preceding case is shown.

| **Human case** | **ID of most closely related case(s)** | **Patristic distance**  **(substitution/site)** | **Estimated divergence (year)** | **Time between cases (day)** |
| --- | --- | --- | --- | --- |
| 1 | KT119642 | 0.0309 | 63.38 | -5447 |
| 2 | OR920307 | 0.0029 | 5.89 | -309 |
| 2 | OR920256 | 0.0070 | 14.41 | +1212 |
| 3 | OR045943 | 0.0034 | 6.92 | -178 |
| 3 | OR920235 | 0.0021 | 4.29 | +758 |
| 4 | OR920236 | 0.0020 | 4.02 | -299 |
| 5 | OR045943 | 0.0027 | 5.47 | -1266 |

Reference

1. Troupin C, Dacheux L, Tanguy M, Sabeta C, Blanc H, Bouchier C, et al. Large-Scale Phylogenomic Analysis Reveals the Complex Evolutionary History of Rabies Virus in Multiple Carnivore Hosts. PLOS Pathog. 2016;12: e1006041. doi:10.1371/journal.ppat.1006041
